# Characterization of a murine model of non-lethal, symptomatic dengue virus infection

**DOI:** 10.1038/s41598-018-22618-w

**Published:** 2018-03-20

**Authors:** Vanessa V. Sarathy, Mellodee White, Li Li, Jaclyn A. Kaiser, Gerald A. Campbell, Gregg N. Milligan, Nigel Bourne, Alan D. T. Barrett

**Affiliations:** 1 Department of Pathology, UTMB, Galveston, TX 77555 USA; 2 Sealy Institute for Vaccine Sciences, UTMB, Galveston, TX 77555 USA; 3 Institute for Human Infections and Immunity, UTMB, Galveston, TX 77555 USA; 4 Department of Pediatrics, UTMB, Galveston, TX 77555 USA; 5 Department of Microbiology and Immunology, UTMB, Galveston, TX 77555 USA

## Abstract

The mosquito-borne disease dengue is caused by four serologically- and genetically-related viruses, termed DENV-1 to DENV-4. Historical setbacks due to lack of human-like mouse models of dengue were partially remedied with characterization of lethal DENV-2 infection in immunocompromised AG129 mice (deficient in IFN-α/β/γ receptors). Recently, our group established lethal AG129 mouse infection models of DENV-1, DENV-3, and DENV-4 using human isolates. Here we compare a non-lethal, disseminated model of DENV-3 infection using strain D83-144 to that of the lethal outcome following infection by strain C0360/94. Both strains belong to DENV-3 genotype II and differ by only 13 amino acids. Intraperitoneal inoculation of AG129 mice with strain D83-144 led to clinical signs of dengue infection, such as cytokine induction, thrombocytopenia, and systemic infection. However, C0360/94 infection led to features of severe human dengue, including coagulopathy and lethal outcome, whereas D83-144 infection does not. This study is the first to investigate a low passage, non-mouse lethal strain in AG129 mice and demonstrates that D83-144 infection induces milder features of human dengue than those induced by lethal C0360/94 infection. The results suggest that the AG129 mouse model has applications to investigate factors associated with mild or severe disease.

## Introduction

Dengue is a mosquito-borne disease that is endemic in most tropical countries and that causes at least 100 million clinical infections each year, including 2.1 million cases of severe dengue fever and 500,000 cases of dengue hemorrhagic fever (DHF)^1^. Dengue is caused by four serologically- and genetically-related viruses, termed DENV-1 to DENV-4, which are members of the genus*Flavivirus*, family *Flaviviridae*. DENV has a single-stranded, positive-sense RNA genome that encodes a single polyprotein, which is processed to generate 10 viral proteins: three structural proteins: capsid (C), membrane (M), and envelope (E); and seven nonstructural (NS) proteins: NS1, NS2A, NS2B, NS3, NS4A, NS4B, and NS5. The E protein comprises the majority of the viral surface and contains antigenic sites targeted by the human immune system^2^.

Clinical dengue infections range in severity from self-limiting, debilitating disease to life-threatening illness^3^. The onset of symptoms occurs following an incubation period of approximately 3–7 days and can include headache, retro-orbital pain, nausea, vomiting, swollen glands, muscle and joint pain, and rash. Disease progression includes severe abdominal pain, hematemesis, and gastrointestinal bleeding. As the viremia wanes and defervescence occurs, individuals may develop severe disease^4^. The pathophysiology of dengue involves vascular leakage, coagulopathies, and thrombocytopenia. DHF and dengue shock syndrome (DSS) are life-threatening and associated with thrombocytopenia, mucosal bleeding, elevated cytokines, vascular leakage, and multi-organ failure.

Coagulopathies during DENV infection include activation of coagulation and fibrinolysis.

Damage to the vasculature leads to the extrinsic pathway of coagulation, which involves tissue factor association with Factor VIIa; subsequent reactions lead to cleavage of prothrombin to thrombin, which catalyzes cleavage of fibrinogen into fibrin monomers, which eventually form fibrin fibrils. Routine tests for hemostasis include prothrombin time (PT), which measures extrinsic pathway factors, and activated partial thromboplastin time (aPTT), which evaluates the intrinsic pathway, thrombin time (TT) and fibrinogen concentration. PT, aPTT, and TT are typically prolonged during DSS^5^.

DENV infections typically resolve and elicit life-long type-specific immunity, but only short-lived cross-protective immunity to the other dengue serotypes^6^. Heterotypic infections are associated with severe disease upon secondary infection; thus, it is important to study the four DENVs and the spectrum of infection.

A major obstacle to the development of vaccines and therapeutics for dengue had been the lack of appropriate animal models that mimic human disease. Dengue is transmitted by *Aedes* mosquitoes to humans in the urban cycle, while the sylvatic (jungle) transmission cycle involves mosquitoes and monkeys^7^. Because DENVs do not naturally infect mice, developing mouse models has been challenging. Advances with immunocompromised mice spearheaded the DENV-2 mouse models^8–10^. Mice deficient in IFN-α/β and IFN-γ receptors (AG129) were infected with a DENV-2 strain adapted by alternate passages between AG129 mice and mosquito cell culture ten times (D2S10) to produce lethal infection with signs of human dengue disease^9^. Subsequently, we established a lethal infection model in AG129 mice utilizing the DENV-3 strain C0360/94, a 1994 Thai clinical isolate that is low-passage and non-mouse adapted^11,12^. Mice developed signs of morbidity, weight loss, systemic infection with high levels of viremia and tissue viral loads, and succumbed to illness 4–7 days post-infection (p.i.). Manifestations of DENV-3 C0360/94 infection included leukopenia, thrombocytopenia, hypoalbuminemia, cytokine storm, vascular leakage into visceral organs but not the brain, and a neutralizing antibody response. Pathologic changes included splenomegaly with immune reactivity and hepatic focal necrosis. Also, we established similar AG129 models using low passage DENV-4 703-4, DENV-4 TVP-376, and DENV-1 WP/74 infection that develop hallmarks of dengue infection in humans^13–15^. Therefore acute, severe, lethal mouse models are available for the four DENVs. However, although humans often recover from dengue infections, all of the symptomatic dengue mouse models cause lethality. Interestingly administering 10-fold lower inoculum of a lethal virus strain into AG129 mice results in transient infection that is non-symptomatic and does not result in hallmarks of dengue^9,12–15^. Specifically, viremia was either undetected or present at lower levels in animals infected with 10-fold less inoculum than in animals infected with lethal-doses of DENV-3 C0360/94, DENV-4 703-4 and DENV-4 TVP-376^12–14^. Taken together, these results indicate that those infections are low-level and asymptomatic. Further, DENV-2 D2S10 and DENV-1 WP/74 infection with 10-fold lower doses did not cause systemic dengue disease, instead led to paralysis^9,15^, indicating that the disease caused by the lower dose is uncharacteristic of dengue disease in humans. One exception to this phenomenon is the D2Y98P model in AG129 mice, which shows lethality over a wide range of doses^10^. In the present study, we characterize mild, non-lethal systemic DENV-3 infection in AG129 mice using a different DENV-3 strain, D83-144, and compare the disease to that caused by strain C0360/94, which leads to lethal infection. Strain D83-144 is non-lethal at 10-fold higher inoculum than C0360/94, and AG129 mice develop disseminated infection, accompanied by cytokine induction and thrombocytopenia, but the disease is less severe than that caused by C0360/94 infection, and mice recover fully. This study shows that the AG129 mouse model can be used to examine factors associated with mild or severe human dengue disease.

## Results

### *In vitro* multiplication kinetics of Thai DENV-3 strains are similar

**Figure 1:**
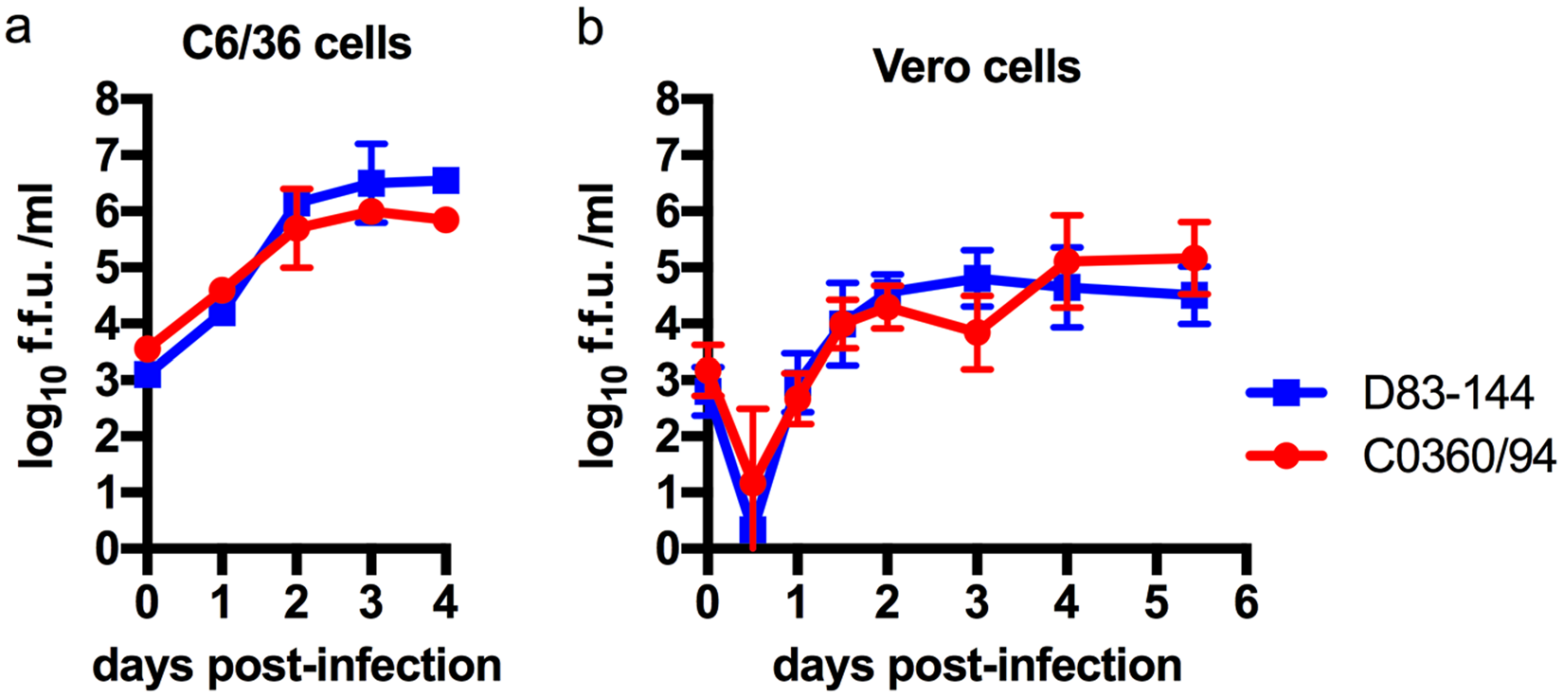
Replication of DENV-3 strains isolated from human cases in Thailand. *In vitro* kinetics of DENV-3 strains at a multiplicity of infection of 0.1 in C6/36 (**A**) or Vero (**B**) cells show that the strains have similar replication. Symbols: mean, error bars: standard error of the mean (SEM), *n* = 3; f.f.u.: focus forming units; limit of detection (L.O.D) 10^2^ f.f.u.

The Thai human isolate D83-144 was selected for study as a low-passage strain that was unable to cause a lethal infection in AG129 mice (see below). D83-144 was prepared as the other DENV strains used in AG129 mouse models, by amplification in C6/36 mosquito cells. Initial studies involved comparing the multiplication kinetics of non-lethal strain D83-144 with lethal strain C0360/94 in C6/36 mosquito cells and monkey kidney Vero cells at a m.o.i. of 0.1. As shown in Fig. 1, strain D83-144 routinely multiplied to the same titer as strain C0360/94 in both C6/36 cells and Vero cells. These results demonstrate that the two strains have similar multiplication kinetics in both arthropod and mammalian cell culture.

### DENV-3 D83-144 is not lethal in AG129 mice

**Figure 2:**
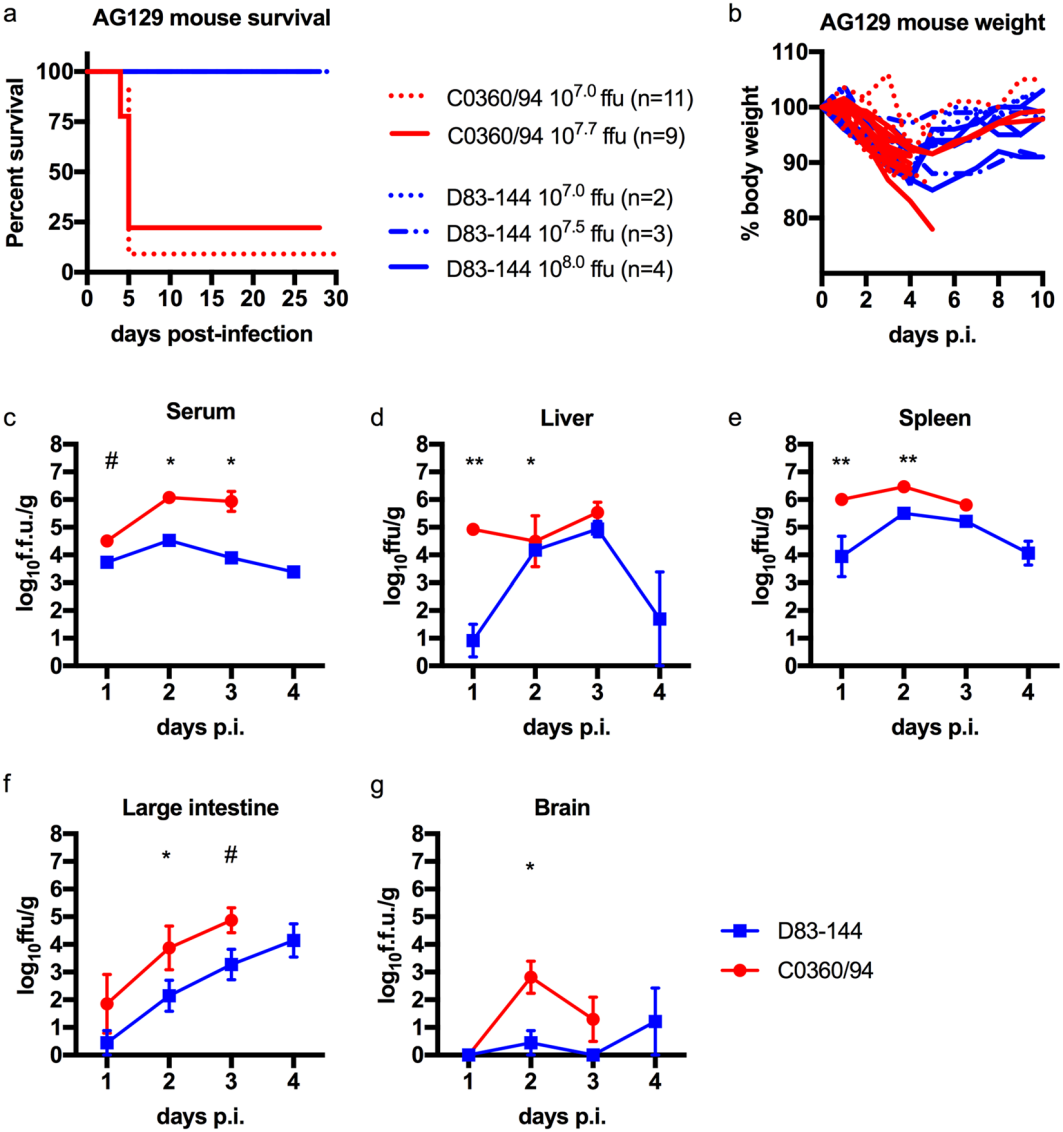
Comparison of DENV-3 disease in adult AG129 mice. (**a**) Kaplan-Meier survival curves of mice infected with 10^7.0^ (*n* = 11) or 10^7.7^ (*n* = 9) f.f.u. of C0360/94 or 10^7.0^ (*n* = 2), 10^7.5^ (*n* = 3), or 10^8.0^ (*n* = 4) f.f.u. of D83-144 via the i.p. route. Mice were monitored for at least 4 weeks. (**b**) Percent weight lost during infection relative to animal weight prior to infection, each line represents the time course of a single animal. (**c–g**) Mice inoculated with 10^7.5^ f.f.u. D83-144 or C0360/94 were sacrificed daily to determine viral loads during acute infection. (c) Blood was collected and serum samples were used for viremia measurement; results are from three independent experiments. Differences in daily viral titers between D83-144 and C0360/94 were determined by Mann-Whitney tests and asterisks denote significance; day 2 *p* = 0.0003, day 3 *p* = 0.0011, day 1 was not significant, *p* = 0.0545 (denoted by #). (**d–g**) Internal organ samples were homogenized, and virus was titrated; results are pooled from two separate experiments. (**d**) Liver viral titers were significantly different on day 1 (*p* = 0.0030) and day 2 (*p* = 0.0466). (**e**) Spleen viral titers were significant on day 1 (*p* = 0.0061) and day 2 (*p* = 0.0012). (**f)** Viral titers in the large intestine were significant on day 2 only (*p* = 0.0344) and trended significant on day 3, *p* = 0.0669 (denoted by #). Viral titers in the brain were low for both D83-144 and C0360/94, but were significantly different on day 2 (*p* = 0.0128). Serum titers are expressed as log_10_ f.f.u./ml and organ titers as log_10_ f.f.u./g of tissue. Symbols represent mean sample titers, error bars: SEM. L.O.D. as follow: serum: 10^2.0^ f.f.u.; liver: 10^2.8^ f.f.u.; spleen: 10^2.8^ f.f.u.; large intestine: 10^3.0^ f.f.u.; brain: 10^2.7^ f.f.u.

AG129 mice were tested for susceptibility to infection with DENV-3 D83-144. Previous studies with DENV-3 C0360/94 and other DENV serotypes showed that inoculation with doses of 10^7.0^ p.f.u and higher were lethal in AG129 mice^12–15^. Therefore, groups (n = 2–4) of six-week-old mice were inoculated intraperitoneally with doses of D83-144 ranging from 10^7.0^ to 10^8.0^ focus forming units (f.f.u.). All mice infected with D83-144 survived challenge (Fig. 2a). To examine clinical signs of infection, animals were monitored daily for four weeks and weight determined daily during acute infection. D83-144-infected mice lost weight starting on day 2 p.i. (Fig. 2b). Mice infected with 10^7.0^, 10^7.5^, and 10^8.0^ f.f.u. of D83-144 exhibited maximum weight loss to 10% (ANOVA, *p* < 0.0001), 7%, and 13% (ANOVA, *p* < 0.0001), respectively, of initial body weight on day 4 then began to gain back weight at day 5 p.i. (Fig. 2b). As positive controls, mice were infected with 10^7.0^ or 10^7.7^ f.f.u of the lethal strain C0360/94. These mice experienced significant weight loss from 2–6 dpi (ANOVA, Dunnett’s post-test, *p* < 0.0001), and subsequently most succumbed to infection as previously observed^12^; because a higher dose would have likely resulted in mortality, C0360/94 infection was not tested at 10^8.0^ f.f.u. inoculum. Importantly, neither DENV-3 strain caused the mice to develop paralysis or signs of neurological disease at any time during the observation period or in any subsequent experiments. These results showed that strain D83-144 did not kill mice even at a dose 10-fold higher than the LD_50_ of strain C0360/94, 10^7.1^ f.f.u., or other AG129-mouse lethal DENVs^12,16^.

To test whether or not the mice had been infected by DENV-3 D83-144, animals were tested for the induction of neutralizing antibodies. At the end of the study (28 days or 52 days), the mice were sacrificed, and terminal bleed sera were harvested to use in focus reduction neutralization titer (FRNT_50_) assays. Animals infected with 10^7.0^ (*n* = 2) or 10^8.0^ (*n* = 4) D83-144 exhibited FRNT_50_ values of 1540 and 1118, respectively, which was comparable to the historical data with C0360/94-infected animals who survived infection: 10^6.5^ f.f.u.: 399, 10^7.0^ f.f.u.: 1279, or 10^7.5^ f.f.u. 634 (Supplementary Table 1)^12^. Together, survival analysis, weight loss monitoring, and antibody neutralization titers provided evidence that AG129 mice developed and survived infection by D83-144. Therefore, a detailed characterization of virus infection was conducted.

### Systemic detection of viral loads in D83-144-infected AG129 mice

DENV-3 D83-144 infection of AG129 mice was characterized and compared to that caused by the lethal strain DENV-3 C0360/94. Because the original characterization of C0360/94 infection was performed with a dose of 10^7.5^ f.f.u. this inoculum was chosen for subsequent studies with both DENV-3 strains^12^. The viral loads in sera and organs were determined following serial sacrifice at 1, 2, 3, or 4 days p.i using focus formation assays (Fig. 2c–g). Mice inoculated with 10^7.5^ f.f.u. D83-144 had detectable viremia (greater than 10^3^ f.f.u./ml) on days 1–4 p.i. and viral load in the spleen on days 1–4 p.i. In the liver and large intestine, D83-144 was either transiently or not detected on day 1 p.i. then on days 2 and 3 p.i., both of these tissues had detectable virus. In the brain, D83-144 was only transiently detected in a few animals. As a positive control, AG129 mice were infected with a lethal dose of 10^7.5^ f.f.u. of C0360/94 (the same dose used for D83-144 infected mice), and mice were sacrificed on days 1-3 p.i. for comparison. Interestingly, viral titers in the serum were approximately 100-fold higher in C0360/94- than in D83-144-infected animals on day 2 (10^4.5^ versus 10^6.1^ f.f.u., *p* = 0.0003 Mann-Whitney) and on day 3 (10^3.9^ versus 10^5.9^ f.f.u., *p* = 0.0011, Mann-Whitney). In comparison, in the tissues, C0360/94 infection consistently led to higher titers than D83-144 infection, with statistical significance on days 1 and 2 in the liver (*p* = 0.0030 and *p* = 0.0426 respectively) and spleen (*p* = 0.0061 on day 1 and *p* = 0.0012 on day 2). In the large intestine, assays showed that titers were significantly different on day 2 (*p* = 0.0344) but not on day 3 (*p* = 0.0669). Further, the low titers caused by C0360/94 in the brain were significantly different from those caused by D83-144 on day 2, although most mice did not have appreciable virus detected in the brain.

The infectivity data demonstrated disseminated viral loads in the serum, liver, spleen, and large intestine of mice infected with strain D83-144, indicative of systemic dengue in AG129 mice. The transient detection of low virus titers in the brain of a small proportion of the animals combined with the absence of neurological signs in mice infected with strain D83-144 for at least 4 weeks p.i. strongly suggested that the animals were not exhibiting neurological disease, which is similar to the results of C0360/94 infection in which mice showed no neurological disease.

### D83-144 infection results in histopathology in AG129 mice

**Figure 3:**
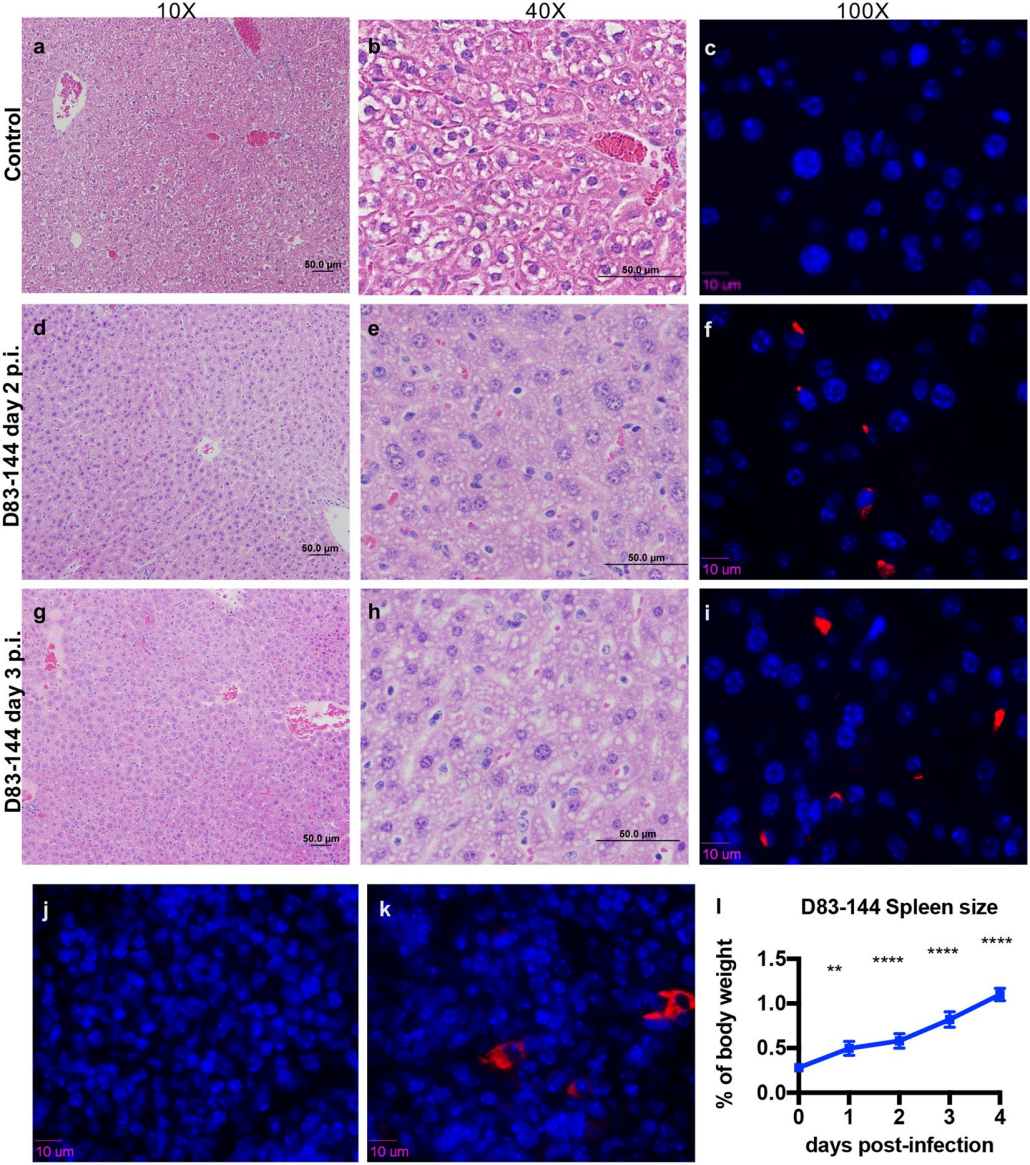
Histopathology during D83-144 infection. (**a–i**) Representative slides from D83-144-infected mice at 2 and 3 days post-infection (p.i.) are shown at 10× and 40× magnification. Controls for comparison are from liver sections from 2 day p.i. mock-infected mice, shown as control (**a**,**b**). At 2 and 3 days p.i. (**d**,**e**,**g** and **h**, respectively) D83-144 infection leads to activation of hepatocytes shown as binucleation or nuclear pleomorphism. To detect active replication in the liver, sections were immunostained with rabbit-anti-DENV-NS3, as described in Methods. Positive (red) signal was observed for DENV NS3 in the D83-144 2 (**f**) and 3 (**i**) days p.i. slides, but not in the control antibody-stained slide (**c**); DAPI was used to stain nuclei (blue). Further, control (**j**) or 2 day p.i, (**k**) spleen samples were stained for NS3 (positive red stain on day 2). Splenomegaly in D83-144 was calculated as a function of spleen mass to total body mass (**g**) and compared with ANOVA and Dunnett’s post-test: day 1 *p* = 0.0029, day 2–4 *p* < 0.0001. Results are from two independent experiments; day 0 and 1, *n* = 4; day 2 and 3 *n* = 7; day 4 *n* = 2.

In order to detect any pathologic effects of D83-144 infection on the organs, tissues from mice inoculated with 10^7.5^ f.f.u. D83-144 were examined for histopathological signs of disease at 1-4 dpi. Other AG129 mouse models of DENV infection are characterized by damage to liver tissues^10,12–15^, and hematoxylin and eosin (H&E) staining of D83-144-infected tissues also revealed histopathological changes in liver sections compared to mock-infected controls (Fig. 3). At day 1 p.i., liver sections appeared quite normal. However, at day 2 p.i., histology showed hepatocyte binucleation and nuclear pleomorphism, which continued to days 3 and 4. Vacuolation was present in several liver sections, and these results indicated that D83-144 infection affected the liver, but not to the extent previously observed during C0360/94 infection (Supplementary Fig. 1). Spleen sections from D83-144-infected mice indicated leukocyte activation and splenic congestion (not shown), and the spleens were enlarged (Fig. 3l) beginning on day 1 (*p* = 0.0029) and progressing though days 2-4 (*p* < 0.0001), as has been shown for all of the DENV AG129 mouse models^12–15^. No cell damage was observed in the intestine sections, and brains from virus-infected mice had no signs of inflammation or damage (not shown). Further, liver sections were immunostained for DENV NS3 antigen (Fig. 3c,f,i), and its presence was detected, indicating that D83-144 replicated in the liver (Fig. 3f,i). Additionally, spleen sections showed similar results with NS3 staining (Fig. 3j,k). Together, these results indicated that D83-144 infection induced gross pathology (splenomegaly) and histopathological changes in AG129 mice.

### D83-144 infection alters AG129 mouse blood chemistry

**Figure 4:**
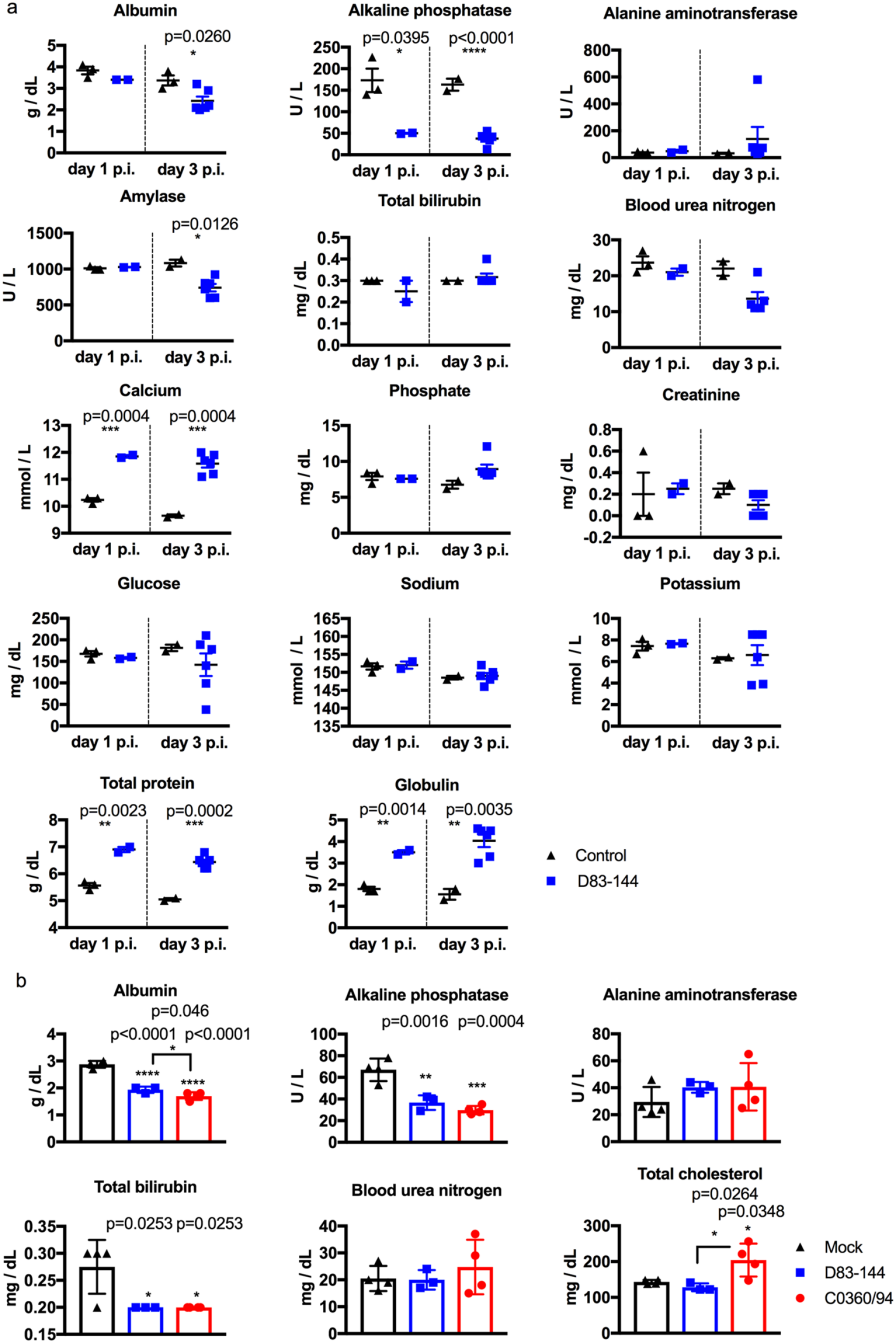
Chemistry profile of D83-144-infected mice. Vetscan was used to analyze Lithium Heparin anticoagulated blood that was harvested from mock- or virus-infected mice sacrificed on day 1 or 3 p.i.; mock: *n* = 3 both days, D83-144: *n* = 2 day 1 and *n* = 6 day 3. (**a**) Comprehensive diagnostic panel rotor analysis of samples. Individual values are represented by symbols and sample means depicted by lines. Asterisks denote significance of D83-144 blood samples compared to uninfected controls using t-tests. Results are pooled from two experiments. (**b**) Samples collected on day 3 after mock (*n* = 4), D83-144 (*n* = 3), or C0360/94 (*n* = 4) infection were analyzed by Mammalian liver profile rotor tests. Bars represent the mean, error bars are SEM, and the three groups were compared using ANOVA with Tukey’s post-test; significant p-values are depicted in the graphs.

To determine the broad effects of D83-144 infection on the blood chemistry of infected AG129 mice, blood was analyzed using Vetscan comprehensive diagnostic profile rotors. Samples harvested from a small group of mice at day 1 (*n* = 2) and at day 3 p.i. (*n* = 6) were compared to mock-infected controls on day 1 (*n* = 3) and 3 (*n* = 3) p.i. (Fig. 4a). D83-144 infection decreased both albumin and amylase levels on day 3 p.i. (*p* = 0.0260 and *p* = 0.0129, respectively). Both days 1 and 3 p.i. blood samples had decreased levels of alkaline phosphatase (*p* = 0.0395 and *p* < 0.0001, respectively), and increased calcium (*p* = 0.0004), total protein (*p* = 0.0023 and *p* = 0.0002 respectively), and globulin (*p* = 0.0004 and *p* = 0.0035, respectively). Unlike previously detected for C0360/94 infection, no imbalance in the blood plasma level of sodium or potassium was detected in D83-144 infected mice^12^. Thus, D83-144 induced some alterations to blood chemistry, but the relative severity was difficult to ascertain. Therefore, a second experiment was conducted using mammalian liver profile rotors (Vetscan) in order to compare the severity of disease, focusing on liver dysfunction, among mock-, D83-144-, and C0360/94-infected mice day 3 post-infection (Fig. 4b). The results of this profile showed that infection with both DENV-3 strains induced some of the same parameters on day 3 p.i., including lower albumin, alkaline phosphatase, and total bilirubin; however, albumin was lower in C0360/94-infected mice (1.8 versus 1.5 g/dL, *p* = 0.046). Further, the C0360/94-infected animals had elevated cholesterol (*p* = 0.0348). Neither DENV-3 strain caused increased levels of liver alanine aminotransferase. Ultimately, the results indicate that AG129 respond to D83-144 infection with hypoalbuminemia and increased globulin and total protein levels, whereas C0360/94-infected mice show those features and electrolyte imbalance of sodium and potassium levels^12^.

### D83-144 infection decreases leukocytes and platelets

**Figure 5:**
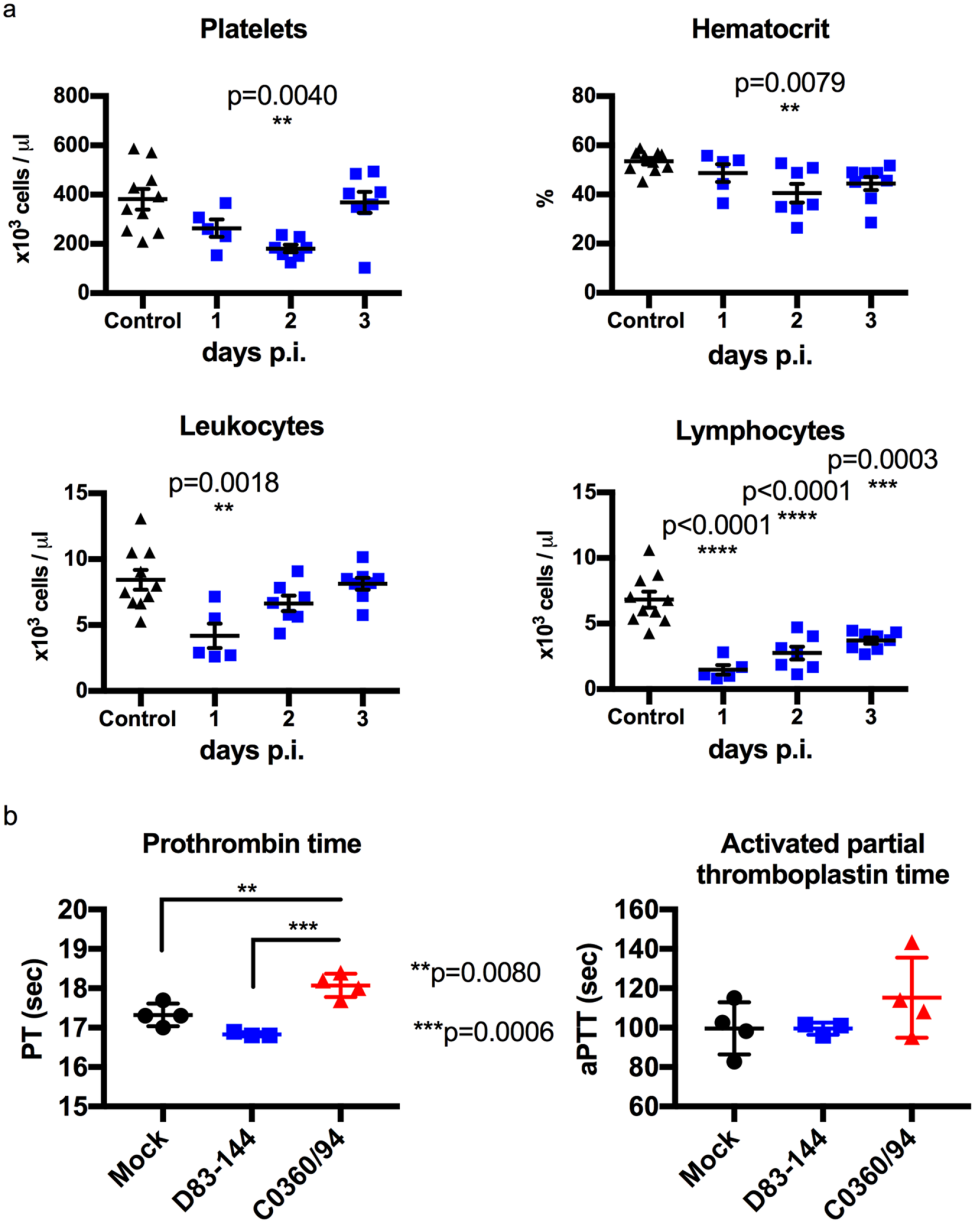
Blood profile of D83-144-infected mice. (**a**) Levels of platelets, percent hematocrit, leukocytes, and lymphocytes were determined from EDTA-anticoagulated blood from control (*n* = 10) or D83-144-infected mice on day 1 (*n* = 5), day 2 (*n* = 7), or day 3 (*n* = 7). Results are pooled from two separate experiments; lines represent the mean daily value, and comparisons between control and infected samples were determined with ANOVA with post-test. (**b**) Effects of D83-144 or C0360/94 infection on coagulation were determined on day 3 p.i. using the Vetscan PT/aPTT test (prothrombin time/activated partial thromboplastin time) on sodium citrate-anticoagulated blood from mock- (*n* = 4), D83-144- (*n* = 3) or C0360/94-infected (*n* = 4) mice.

The ability of non-lethal D83-144 infection to affect blood cell counts of AG129 mice was examined. Blood samples from uninfected (n = 10) or D83-144-infected mice were collected at 1 (n = 5), 2 (n = 7) and 3 (n = 7) days p.i. Complete blood counts showed that, compared to uninfected controls, D83-144 lowered platelets (*p* < 0.0040) and a small decrease in hematocrit (*p* < 0.0079) was detected on day 2 p.i (Fig. 5a). Effects on leukocyte numbers were present earlier, on day 1 p.i., including sharp decrease in lymphocytes on days 1 (*p* = 0.0003), 2 and 3 p.i. (both *p* < 0.0001). Because both viruses D83-144 and C0360/94 lead to lower numbers of platelets, coagulation was tested in mice by determination of the prothrombin (PT) and thromboplastin times (aPTT). Mock, D83-144, or C0360/94-infected animals were sacrificed on day 3 p.i., and blood was tested using a combination PT/aPTT test (Fig. 5b). Results showed that animals infected with C0360/94 had a statistically significant increase in prothrombin time compared to mock-infected (*p* = 0.0080) and D83-144-infected (*p* = 0.0006) mice. However, no statistical significance was detected in aPTT. Together blood tests determined that D83-144 infection leads to lymphopenia and acute thrombocytopenia, which are signs of human dengue detected during C0360/94 infection, but no changes in PT or aPTT, the former which was prolonged during lethal C0360/94 infection.

### D83-144 infection results in elevated cytokine expression in AG129 mice

**Figure 6:**
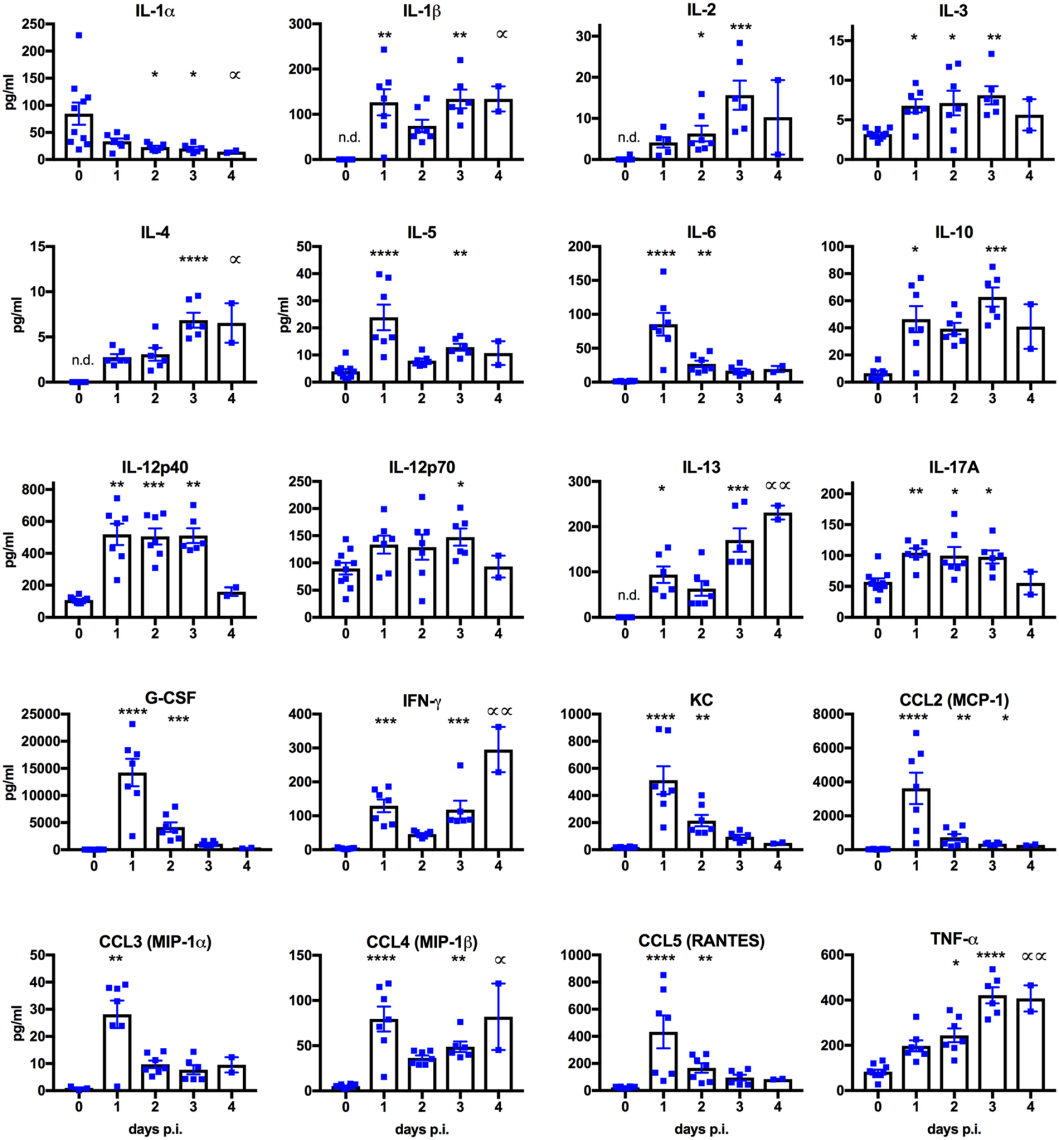
D83-144 infection with 10^7.5^ f.f.u. leads to altered serum cytokine levels. Sera harvested from naïve (*n = *10) or D83-144-infected mice (day 1 *n* = 7, day 2 *n* = 6, day 3 *n* = 7, day 4 *n* = 2) were analyzed using Bioplex. Bars represent the mean; error bars are the SEM. Daily changes in values of infected samples for 1-4 dpi were compared using Kruskal-Wallis ANOVA with post-test, and statistical significance is depicted with asterisks above the columns as follow: ****p < 0.0001, ***0.0001 < *p* < 0.001, **0.001 < *p* < 0.01, *0.01 < *p* < 0.05. For day 4 p.i. group, significance is denoted by a distinct symbol (∝) to highlight that analyses were from *n* = 2; some control values were not determined (n.d.) for some cytokines because the levels were undetectable.

Altered expression of several cytokines has been observed in human samples from patients diagnosed with DF or DHF/DSS^17,18^ and in other mouse models of infection^10,12–15^. In order to evaluate D83-144 induction of cytokine expression, sera were harvested from controls or infected mice at 1–3 days p.i. and analyzed using Bioplex. Kruskal-Wallis tests with multiple comparisons post-tests were performed to detect changes in cytokine values between days (Fig. 6). Three cytokines were elevated on days 1–3 p.i.: IL-3, IL-12p40, and IL-17A. Other cytokines including some hallmarks of dengue infection, peaked on day 1, then subsided: IL-5, IL-6, G-CSF, KC, CCL2, CCL3, and CCL5; while others were elevated on day 1, decreased on day 2, then rose again on day 3: IL-1β, IL-10, IL-13, IFNγ, and CCL4. Interestingly, only three cytokines increased in levels throughout duration of infection: IL-2, IL-4, and TNFα, the latter being important for DENV-3 C0360/94 infection of AG129 mice^12^. Unlike the lethal AG129 models, though, IL-1α decreased in response to D83-144 infection. Lastly, three cytokines were not analyzed due to low numbers of values, which are not suitable for statistics (IL-9, GM-CSF, Eotaxin). Further, some of the evaluated cytokines are not typically present in uninfected mouse serum, and resulted in insufficient numbers of naïve mouse samples for comparison; to those groups, a value of ‘0’ was added until there were *n* = 6 values to perform statistics with the 1–4 days p.i. groups. Together the Bioplex analysis showed that D83-144 elicits a cytokine and chemokine response.

### Molecular analysis reveals changes between AG129-lethal and non-lethal DENV-3 strains

**Figure 7:**
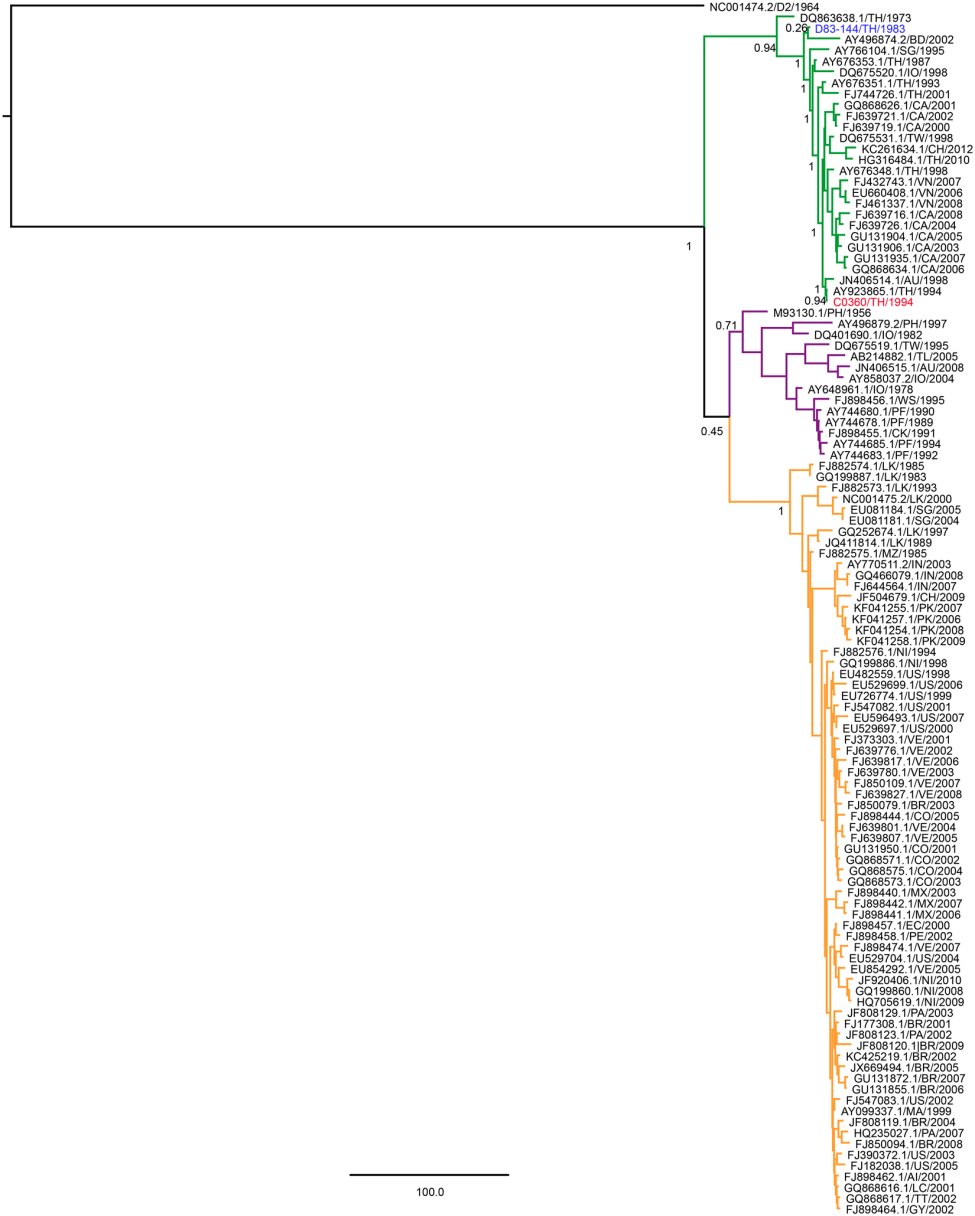
Bayesian phylogenetic analysis of nucleotides 38-10606 of representative strains from DENV-3 genotypes I (purple clade), II (green clade) and III (orange clade), and DENV-2 16681 (outgroup). Posterior probabilities of major nodes are included. C0360/94 and D83-144 are in red and blue, respectively. Strains are shown with accession number, country, and year of isolation. When multiple strains were available, only one strain per country per year was included in the analysis. Country abbreviations: Anguilla (AI), Australia (AU), Bangladesh (BD), Brazil (BR), Cambodia (CA), China (CH), Colombia (CO), Cook Islands (CK), East Timor (TL), Ecuador (EC), French Polynesia (PF), Guyana (GY), India (IN), Indonesia (IO), Martinique (MA), Mexico (MX), Mozambique (MZ), Nicaragua (NI), Pakistan (PK), Paraguay (PA), Peru (PE), Philippines (PH), Samoa (WS), Saint Lucia (LC), Singapore (SG), Sri Lanka (LK), Taiwan (TW), Thailand (TH), Trinidad and Tobago (TT), United States of America-Puerto Rico (US), Venezuela (VE), Vietnam (VN).

In order to identify potential molecular determinants of virulence associated with lethal DENV-3 disease in AG129 mice, both the lethal C0360/94 and non-lethal D83-144 genome sequences were examined. Bayesian phylogenetic analysis of representative DENV-3 strains indicated that both strains belong to genotype II with other Thai and southeast Asian strains but are distantly related within the genotype (Fig. 7). Sequence alignments between the two strains revealed 108 nucleotide differences, including four in the 3′ UTR (Table 1). The nucleotide differences encoded 13 amino acid changes: five in the structural proteins (one in M and four in E) and eight in the nonstructural proteins (four in NS2A, one in NS3, one in NS4B and two in NS5) (Table 1).

**Table 1:** Coding and 3′ UTR changes between the D83-144 and C0360/94 genomes.

Nucleotide	Polyprotein	Protein	D83-144	C0360/94	Location description
932	280	M 75	A	T	Cleavage site at M-E junction
1304	404	E 124	S	P	E domain 2
1328	412	E 132	H	Y	E domain 2
1448	452	E 172	I	V	E domain 1
2370	759	E 479	A	V	E transmembrane domain
3866	1258	NS2A 133	A	T	pTMS5
4007	1305	NS2A 180	V	L	pTMS7
4052	1320	NS2A 195	A	T	pTMS8
4235	1340	NS2A 215	L	P	Carboxy-terminus in ER lumen
5709	1872	NS3 399	K	R	Helicase: domain 2
7560	2489	NS4B 247	K	R	Cleavage site at the NS4B-NS5 junction
8577	2828	NS5 339	I	T	RNA-dependent RNA polymerase (RdRp): α4 helix of the beta-Nuclear localization site (βNLS)
8730	2879	NS5 389	R	K	RdRp: between the α6 and α7 helices of α/βNLS
10271	3′ UTR	—	g	a	—
10288	3′ UTR	—	g	a	—
10391	3′ UTR	—	u	g	—
10392	3′ UTR	—	u	a	—

**Figure 8:**
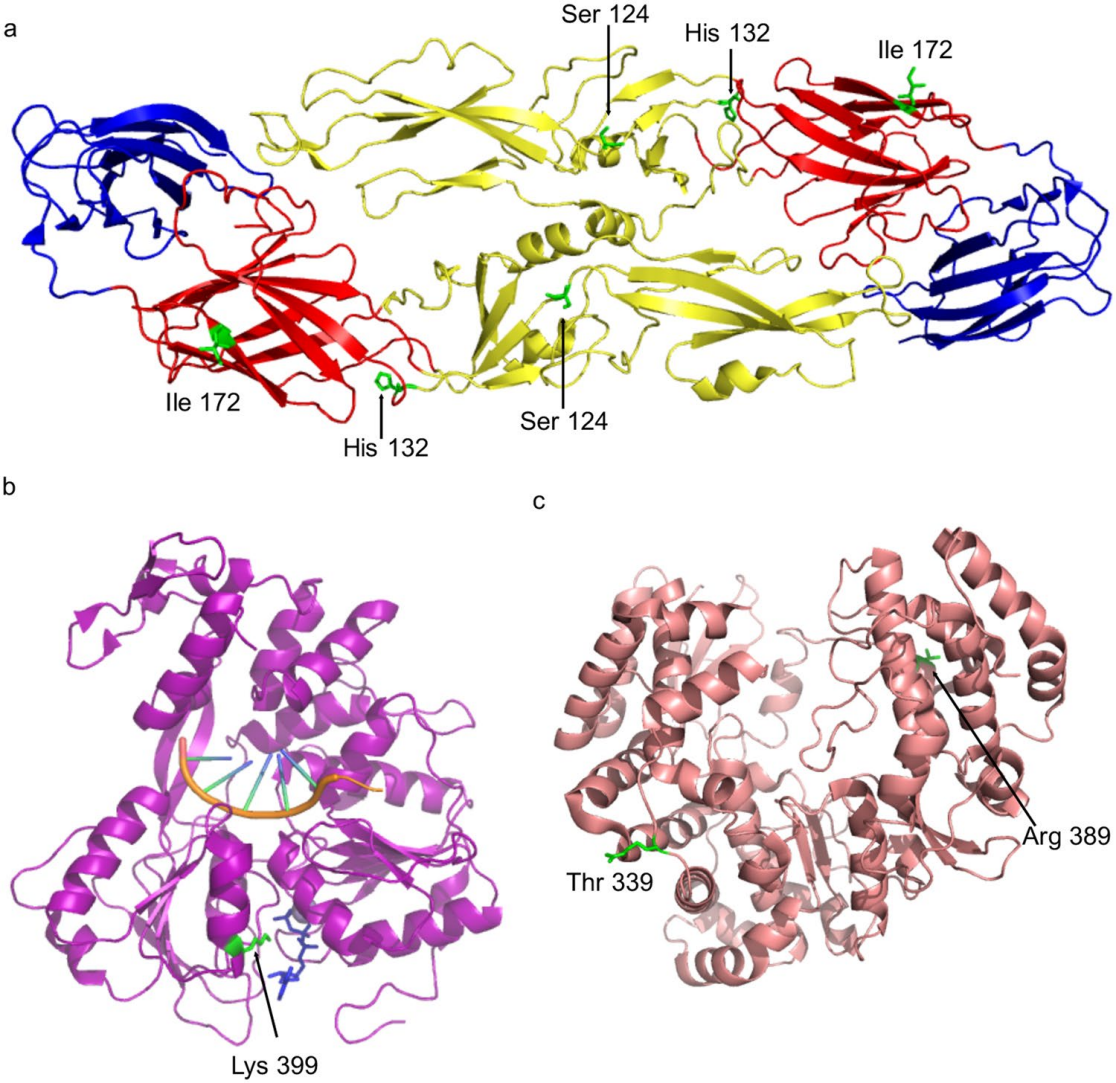
Location of potential molecular determinants of DENV-3 based on solved structures in E protein, NS3 Helicase, and RNA dependent RNA polymerase. (**a**) The E protein ectodomain homodimer cartoon is shown with ED1 in red, ED2 in yellow, and ED3 in blue. The D83-144 amino acids are depicted as sticks in green: Ser 124, His 132, and Ile 172. (**b**) The DENV-4 NS3 Helicase protein cartoon is shown in purple with a nucleic acid (orange), a nonhydrolysable ATP analogue, AMPPNP, in blue, and Lys 398 (equivalent to DENV-3 D83-144 Lys 398) as a stick in green. (**c**) The DENV-3 NS5 RNA dependent RNA polymerase cartoon is shown in salmon with the C0360/94 amino acid Thr 339 and D83-144 amino acid Arg 389 in green sticks.

Amino acid differences between the structural proteins of D83-144 and C0360/94 were examined by alignment to sequences of solved virus protein structures. The M protein A75T difference is the last residue in the protein, which is located in the conserved signalase cleavage site at the junction of M-E proteins, and flaviviruses have been shown to contain both alanine and threonine at this position^2^. Three of the E protein changes are denoted in the E dimer structure, which depicts the residues encoded by D83-144 at those positions (Fig. 8a). E S124P, E H132Y, and E I172V are surface-exposed and lie in the EDII central interphase, the EDI-II interphase, and EDI, respectively. E A479V localizes to the E transmembrane domain, and is therefore not included in the E ectodomain structure. Interestingly, all four E changes identified between D83-144 to C0360/94 occur in positions previously identified as DENV-3 informative/variable sites^19^. Furthermore, H132Y, and to a lesser extent, S124P may lead to a local loss of positive charge. Significantly, basic amino acids in EDII, specifically N124D and K128E mutations have been implicated in the virulence of DENV-2 strains in the AG129 model^20^.

Coding differences in the nonstructural proteins NS3 and NS5 were analyzed using the solved helicase and RNA-dependent RNA polymerase structures. The NS3 K399R change is located in domain 2 of the helicase (Fig. 8b) and is not predicted to be involved in interdomain interaction, ATP binding, or RNA binding^21^. Both NS5 changes reside in the nuclear localization sites (NLS) of the RNA-dependent RNA polymerase^22^: I339T lies in the α4 helix of the βNLS, and K389R is in the α/βNLS between the α6 and α7 helices (Fig. 8c). The changes in DENV-3 NS2A and NS4B (data not shown) were mapped by comparison to the equivalent residues in the DENV-2 transmembrane topologies^23,24^. NS2A A133T, V180L, and A195T have predicted locations in pTMS5, pTMS7, and pTMS8, and L215P is in the carboxy-terminus of NS2A in the ER lumen. The NS4B K247R is the penultimate residue in this protein, which is predicted to translocate from the cytoplasm to the ER lumen following viral protease cleavage of NS5^24^.

In summary, thirteen amino acid differences between D83-144 and C0360/94 were identified, one or more of which may contribute to the virulence of C0360/94 or attenuation of D83-144 in AG129 mice, especially, E 124 and E 132.

## Discussion

Lethal models of primary dengue infection now exist for DENV-1 strain WP/74; DENV-2 strains S10, D220, S221, and D2Y98P; DENV-3 strain C0360/94, and DENV-4 strains 703-4 and TVP-376 that mimic several aspects of human dengue in immunocompromised AG129 mice^9,10,12–16,25–27^. Further, ADE-mediated lethal disease has also been successfully modeled by the low passage clinical isolates DENV-1 2402 and DENV-2 3295^28,29^. Infection with approximately 10^7.0-7.5^ f.f.u. results in development of acute, symptomatic infections with features such as viremia, leukopenia, thrombocytopenia, hypoalbuminemia, plasma leakage, organ pathology, and cytokine storm^12–15^. Utilization of the same virus strains at sub-lethal doses, 10-fold less, typically results in asymptomatic, transient infection^12–14^. In the present study, we identified that infection of AG129 mice with DENV-3 D83-144, which is phylogenetically-related to DENV-3 C0360/94 and has similar multiplication kinetics in mammalian and mosquito cell culture, led to many of the features of dengue in AG129 mice, yet the mice all survived. The present study is the first report of a sub-lethal dengue model in AG129 mice in which animals develop disseminated disease in the absence of neurologic signs or enhancement.

Detailed characterization of AG129 mice infected with strain D83-144 showed that there were many similarities to the previously reported lethal AG129 mouse models^9,10,12–15^; however, D83-144 infection was less severe. Table 2 summarizes the similarities and differences between the lethal and non-lethal DENV-3 -genotype II infections. Because the lethal DENV-3 strain C0360/94 has an LD_50_ of 10^7.1^ f.f.u. in AG129 mice, doses up to 10^8.0^ f.f.u. of D83-144 were used to test for lethality. However, even at this high dose, D83-144 did not cause lethality, indicating that either a much higher dose of D83-144 is needed to achieve lethality or that D83-144 non-lethal disease is not dose-dependent in the AG129 mouse model. One of the challenges of DENV mouse models has been the ability to mimic human disease. Infection with D83-144 induced hallmarks of dengue, such as pro-inflammatory cytokines, thrombocytopenia, lymphopenia, and organ involvement. However, the animals had milder symptomatic infection characterized by transient weight loss without additional morbidity, such as ruffled fur, hunched posture, or limited mobility. Like C0360/94, D83-144 did not result in appreciable brain viral loads or any paralysis.

**Table 2:** Comparison of DENV-3 strains utilized in the AG129 mouse model.

	D83-144	C0360/94
Isolation	Thai human infection 1983	Thai human infection 1994
*In vitro* Vero titer (f.f.u.)	10^5^	10^5^
*In vitro* C6/36 titer (f.f.u.)	10^6^	10^6^
Morbidity	weight loss, then recovery	**hunching, decreased mobility**, weight loss
Neurological disease	None	None
Mortality	No	**Yes**
Viremia (f.f.u.)	10^4.5^	**10** ^**6**^
Liver viral titer (f.f.u.)	10^5^	10^5.5^
Spleen viral titer (f.f.u.)	10^5.5^	10^6.5^
Intestine viral titer (f.f.u.)	10^4^	10^5^
Liver histopathology	Nuclear pleomorphism, binucleation, hepatocyte vacuolation, viral replication in Kupffer cells	Nuclear pleomorphism, binucleation, hepatocyte vacuolation, **focal necrosis**, viral replication in Kupffer cells
Spleen pathology	Leukocyte activation, splenic congestion, splenomegaly, viral replication	Leukocyte activation, splenic congestion, splenomegaly, viral replication, destruction of splenic architecture
Leukopenia	Yes	Yes
Thrombocytopenia	Yes	Yes
Hypoalbuminemia	Yes	**Yes, more severe**
Electrolyte imbalance	No	**Yes**
Prolonged prothrombin time (PT)	Not significant	**Yes**
Activated partial thromboplastin time (aPTT)	Not significant	Not significant
Cytokine induction	Low induction: IL-6, IL-10, IL-12, IL-13, IL-17A, G-CSF, IFNγ, TNFα	**High induction**: IL-6, IL-10, IL-12, IL-13, IL-17A, G-CSF, IFNγ, TNFα
Chemokine induction	Low induction: KC, CCL2, CCL3, CCL4, CCL5	**High induction**: KC, CCL2, CCL3, CCL4, CCL5

Characteristics of increased severity of C0360/94 infection compared to D83-144 are bolded.

Comparison with lethal dose-C0360/94 infection showed that D83-144 exhibited reduced replication in mouse tissues, especially in the serum and large intestine (Fig. 2c and f), although no multiplication deficiencies were detected in cell culture passaging *in vitro*. Next, tissue sections from infected animals were examined for histopathological changes. Overall, D83-144 infection caused many of the histopathological changes observed during C0360/94 infection, but the changes were not as severe, and did not include hepatic necrosis. The levels of splenomegaly were similar, indicating that the splenic enlargement and congestion were equivalent during both non-lethal and lethal DENV-3 symptomatic infections^12^. Although not shown, it is worth noting that D83-144-infected mice had no observable ascites or effusions during necropsy. This is in contrast to C0360/94-infected animals, which, during necropsy, typically present effusions, ascites, bloating, and signs of diarrhea.

Along with organ involvement, a comprehensive blood chemistry analysis showed that D83-144 leads to decreased alkaline phosphatase and amylase levels, which can indicate dysfunction of the liver or intestines, and in the kidneys. Also, decreased albumin and increased calcium were both detected, as had been the case for lethal C0360/94 infection^12^; although albumin was decreased in both DENV-3 infections, hypoalbuminemia was more severe during C0360/94 infection (*p* = 0.046). In accordance with the lack of morbidity and diarrhea, there were no effects on potassium or sodium levels during D83-144, which are both affected by C0360/94^12^. Further examination of blood from infected animals revealed that thrombocytopenia and leukopenia were detected after D83-144 infection, similar to the other systemic AG129 mouse models of dengue. Interestingly, prolonged PT was only detected during C0360/94 infection, indicating a differential coagulopathy preceding lethal infection with C0360/94 that did not occur during non-lethal D83-144 infection.

The cytokine profile data indicate that infection with D83-144 leads to innate immune response changes similar to those of other mouse models, and importantly to those of humans with dengue. Numerous mouse infection and human sample studies have examined the acute cytokine response to DENV^9,18,30–32^. Bioplex analysis of lethal-dose D83-144-infected animals showed that chemokines, inflammatory cytokines, and growth, proliferation, and differentiation cytokines had statistically significant elevated levels compared to uninfected controls (Fig. 6). Several of these cytokines have been identified as markers of dengue in humans, including IL-12, IL-13, IL-17A, G-CSF, IFNγ, and TNFα. However, the levels of cytokines are higher during C0360/94 infection (Table 2)^12^. Furthermore, TNFα has been specifically implicated as contributing to the disease progression of AG129 mice infected with DENV-2 strain S10 and D2Y98P and DENV-3 C0360/94^9,12^. Interestingly, lethal C0360/94 infection causes induction of TNFα as early as 1 day p.i., which is maintained between 600–800 pg/ml on all days p.i. (1–4); however, induction of TNFα following D83-144 infection is gradual and reaches its maximum level at day 3 (400 pg/ml). This difference in cytokine induction may affect the overall virulence of the D83-144 strain *in vivo*.

Markers of DENV-3 infection severity have been previously evaluated^33^. The most resounding results of these analyses is the relationship between disease severity and viremia, which corresponds with the data in this report. Approximately a 100-fold difference in viremia was detected between C0360/94 and D83-144 in AG129 mice, which is similar to the comparison of DENV-3 levels in the plasma of dengue fever versus DHF grade II patient samples (approximately 10^1.5^ difference)^33^. Secondly, both IL-10 and IFNγ were more elevated during severe C0360/94 infection at 800 pg/ml and 1,200 pg/ml, respectively, compared to the 60 pg/ml and 250 pg/ml, respectively, during D83-144 infection. Indeed, during DHF grade II, maximum levels of IL-10 and IFNγ doubled. Therefore, the data gathered from D83-144 and C0360/94 infection are in accordance with the literature on mild and severe DENV-3 infections.

In the AG129 mouse, C0360/94 and D83-144 infection both led to dengue. However, the ultimate phenotype of the two strains was different, as C0360/94 was lethal, and D83-144 was not. Genomic analysis was performed in an effort to understand the molecular basis of virulence differences between the non-virulent and virulent DENV-3 strains. D83-114 and C0360/94 differ by 13 amino acids in the polyprotein (Table 1). The most interesting differences were observed in the E protein at residues S124P and H132Y. Both amino acids were previously identified as potential variable sites in DENV-3, but were not the focus of that study^19^. A nearby residue, E126K, was shown to confer virulence onto a DENV-3 intertypic chimeric virus in a suckling mouse model of infection^34^. Furthermore, S124 and H132 are located in the same region as the DENV-2 residues N124D and K128E, which have been implicated in the mechanism of disease in AG129 models^20^. In the DENV-2 D2S10 model of infection, the strain virulence is attributed to the loss of basic residues (asparagine and lysine) that mediate binding to host heparan sulfate, thereby evading viral clearance^20^. The E S124P (polar to nonpolar) and E H132Y (positive to nonpolar) substitutions may contribute to the virulence of C0360/94 and attenuation of D83-144 by a similar mechanism. Analysis of these strain differences could provide further information into the interaction between the virus and the host.

Several different DENV-3 strains have been used to infect mice in the past. Some models focused on characterization of brain infection or used a brain-adapted DENV-3^35,36^. Intracranial infection of C57BL/6 mice with a DENV-3 genotype I strain caused meningitis, while genotype III was asymptomatic. In later studies with C57BL/6Ja (and knockout mice), a DENV-3 genotype I strain was brain-adapted in order to cause lethal, non-neurotropic disease in mice, and the authors focused on deciphering immune mechanisms of disease^36^. In comparison, this study shows that two low passage, non-adapted, non-neurotropic genotype II DENV-3 strains, D83-144 and C0360/94, have been found to cause human-like dengue in AG129 mice. However, strain-specific differences in C0360/94 may explain lethality of infected mice. Examination of larger panels of low-passage isolates in the AG129 mouse model could provide detailed information on the virulence phenotype of DENV-3.

## Materials and Methods

### Cell culture

Monkey kidney Vero cells were maintained at 37 °C in 5% CO_2_ in minimum essential media (MEM) supplemented with 2 mM L-glutamine, 0.1 mM non-essential amino acids, 100 U/ml penicillin – 100 μg/ml streptomycin, and 8% bovine growth serum (BGS). C6/36 mosquito cells were maintained at 28 °C in MEM supplemented with 2 mM L-glutamine, 0.1 mM non-essential amino acids, 100 U/ml penicillin – 100 μg/ml streptomycin, 1 mM sodium pyruvate, tryptose phosphate buffer, and 10% fetal bovine serum.

### Virus

DENV-3 strains were acquired from the World Reference Center of Emerging Viruses and Arboviruses (WRCEVA) at the University of Texas Medical Branch (UTMB). Strain D83-144 was isolated from a clinical DEN case in Thailand in 1983, and strain C0360/94 was also isolated from a clinical case and has been described recently^11,12^. Seed stocks of both viruses were amplified 2-4 times in C6/36 cells with 2% FBS. For AG129 infection, virus was harvested and concentrated using a 100 K MWCO Amicon at 3000 rpm, 4 °C, for 20 minutes, then quantified by focus assays in Vero cells. Briefly, cells in 12-well plates were infected with 10-fold virus dilutions for 30 minutes before overlay with MEM containing 2% FBS-0.8% carboxymethyl cellulose and incubated for five days at 37 °C. Wells were fixed with acetone:methanol, and foci were visualized by immunostaining with the pan-mosquito-borne flavivirus monoclonal antibody 4G2 or DENV-3 specific mouse immune ascitic fluid. Virus titers are expressed as f.f.u./ml.

### Mouse infection

AG129 mice were bred and maintained at the UTMB. Experiments were approved by the UTMB Institutional Animal Care and Use Committee and performed according to institutional guidelines. Male and female mice 6–12 weeks old were inoculated by intraperitoneal injection with different doses of D83-144 or C0360/94. Animals were weighed and the course of disease monitored until 30 days post-infection (p.i.). The survival analysis includes mice that were euthanized because they exhibited overwhelming signs of severe disease or weight loss (below 80% of initial body weight). To characterize disease, groups of 3-4 mice were infected with 10^7.5^ f.f.u. D83-144 and either sacrificed at 1–4 days post-infection (dpi) or left for outcome observation to be used in survival, morbidity, and weight loss (Fig. 1). For comparison, mice were infected with 10^7.5^ f.f.u. C0360/94. Mock infections consisted of 0.1 ml of cell-conditioned media from C6/36 cells. At time of sacrifice, blood, liver, spleen, brain, and large intestine samples were harvested.

### Mouse necropsy

Following infection with 10^7.5^ f.f.u. D83-144, groups of 3–4 mice for two independent experiments were sacrificed at 1–4 dpi. Sera were harvested from blood in serum separator tubes. Spleens were weighed to assess splenomegaly in the infected animals. To determine viral loads, organ samples were collected into pre-weighed tubes containing triple-pure zirconium beads, and tissues were homogenized using a BeadBug (Benchmark Scientific). Titers were determined using focus-formation assays and were expressed as f.f.u. per gram of tissue or milliliter of serum.

### Histology

At the time of necropsy, liver, spleen, large intestine, and brain samples were harvested and immediately fixed in 10% neutral-buffered formalin. Tissues were paraffin embedded, sectioned, and stained with hematoxylin and eosin (H&E) at the UTMB Research Histopathology Core Laboratory. Slides were analyzed for histopathologic changes as previously described^12^.

### Fluorescence immunostaining

Paraffin-embedded sections from control or infected AG129 mice were immunostained for non-structural protein 3 (NS3) with rabbit polyclonal anti-DENV NS3 (Genetex GTX124252) diluted 1:100, followed by detection with goat anti-rabbit IgG F(ab’)2–Alexa Fluor 594 (Molecular Probes) diluted 1:200, and mounting with Vectashield mounting medium containing DAPI (Vector Laboratories). As controls, sections from mock-infected animals were stained with anti-NS3, and infected sections were stained with rabbit IgG (isotype control).

### Blood chemistry

Blood was collected from control or D83-144-infected mice in Lithium Heparin anticoagulant microtainer tubes, and 0.1 ml of each sample was analyzed for using Vetscan comprehensive diagnostic profile rotors (Abaxis) on days 1 and 3 p.i. A second study of day-3 infection samples from mock, C0360/94-, or D83-144-inoculated AG129 mice was conducted using Vetscan mammalian liver profile rotors (Abaxis).

### Complete blood count

Blood from control or infected AG129 mice was collected into EDTA anticoagulant tubes and analyzed for blood counts using a Hemavet 950TS (Drew Scientific) as previously described^12^.

### Prothrombin time (PT) and activated partial thromboplastin time (aPTT)

Blood was collected into sodium citrate anticoagulant tubes at a 1:9 ratio. Samples were tested in the PT/aPTT test sticks in a Vetscan VSpro analyzer (Abaxis).

### Cytokine analysis

The Bio-Rad Bio-Plex Pro Mouse Cytokine 23-plex was used to test cytokine levels in 12.5 μl of serum according to manufacturer’s instructions. Sera collected from mice 1–4 dpi were analyzed and compared to controls using Kruskal Wallis non-parametric tests. Statistical analyses are described in the figure legend. Cytokine abbreviations: interleukin (IL), keratinocyte chemoattractant (KC), granulocyte-colony stimulating factor (G-CSF), granulocyte/macrophage-colony stimulating factor (GM-CSF), interferon gamma (IFNγ), tumor necrosis factor alpha (TNFα), chemokine ligand (CCL), MCP-1: monocyte chemoattractant protein-1 (CCL2), MIP-1α/β: macrophage inflammatory protein (CCL3/4), RANTES: regulated on activation, normal T cell expressed and secreted (CCL5).

### Virus neutralization

Mouse antibody response was tested using focus reduction neutralization titration (FRNT_50_) assays. Briefly, sera were harvested at 29 or 52 dpi from terminal mouse bleeds and heat-inactivated. Two-fold serial serum dilutions were incubated with 50 f.f.u. of virus for one hour at room temperature and subsequently used to infect Vero cells in 12-well plates for virus titration using focus-formation assays. The titers were analyzed using the log(inhibitor) vs. normalized response–variable slope non-linear regression model in GraphPad Prism v6.0 to acquire the FRNT_50_ and 95% confidence interval.

### Molecular analysis

Viral genome consensus sequences (C0360/94: KJ737429, D83-144: KJ737430) were aligned with ClustalW^37^ and viewed in Seaview^38^ to determine the sequence differences of the two strains. Pymol renderings of the E (PDB 1UZG), NS3 Helicase (PDB 2JLV), and NS5 RNA Polymerase (PDB 2J7U) were used to map the amino acid differences between D83-114 and C0360/94.

### Phylogenetic analysis

Genomic sequences were downloaded from Genbank. Sequences without collection dates or location and of recombinant viruses were omitted, and only one strain per country per year of isolation was included. MUSCLE alignments of sequences corresponding to nucleotides 37-10606 were performed using the CIPRES Science Gateway. Bayesian-inferred coalescent phylogenetic trees were generated using BEAST v1.8 package on CIPRES using the GTR + I + Γ_4_ substitution model with an exponential molecular clock and an exponential distribution. 100 million states were down-sampled with a 3% ‘burn-in’ using Tracer v1.5. Trees were visualized and edited with FigTree v1.4. DENV-2 16681 was used as an outgroup in phylogenetic comparisons.

### Statistical analyses

Neutralization curves were normalized using Microsoft Excel. All bar, scatter, XY, and line graphs, as well as, statistical analyses (mean, median, standard error, t test, Mann-Whitney U test, Kruskal-Wallis ANOVA and post-test, and non-linear regression) were generated with Graphpad Prism v6.0. Statistical significance depicted in figures: ****p < 0.0001, ***0.0001 < *p* < 0.001, **0.001 < *p* < 0.01, *0.01 < *p* < 0.05, ^#^0.05 < *p* < 0.99. Other symbols are described in the figure legends.

### Data availability

The data supporting the findings of this study are included in this article and its Supplementary Data file.

## Electronic supplementary material

Supplementary data
