# Supplementary material for: Characterization of a murine model of non-lethal, symptomatic dengue virus infection
Source: Sci Rep. 2018 Mar 20;8:4900. doi: 10.1038/s41598-018-22618-w (PMC5861036; doi:10.1038/s41598-018-22618-w)
Supplement: Supplementary file 1 — Supplementary data [file 41598_2018_22618_MOESM1_ESM.docx]

**Characterization of a murine model of non-lethal, symptomatic dengue virus infection**

Vanessa V. Sarathy, Mellodee White, Li Li, Jaclyn A. Kaiser, Gerald A. Campbell, Gregg N. Milligan, Nigel Bourne, and Alan D.T. Barrett

Supplementary Table 1: FRNT_50_ values of six-week-old AG129 mice infected with DENV-3.

|  | D83-144 | | C0360/94 | | |
| --- | --- | --- | --- | --- | --- |
| Inoculum | **10^7.0^** | **10^8.0^** | **^10^6.5^** | **10^7.0^** | **^10^7.5^** |
| *n* | 2 | 4 | 8 | 1 | 1 |
| FRNT_50_  (95% CI) | 1540  (1140-2082) | 1118  (905-1379) | 399  (338-472) | 1279  (1077-1518) | 634  (546-736) |
| *r*^2^ | 0.90 | 0.91 | 0.88 | 0.96 | 0.99 |
| dpi | 52 | 28 | 29 | 52 | 29 |

^Reported in Sarathy, 2015^12^.

Supplementary Figure 1


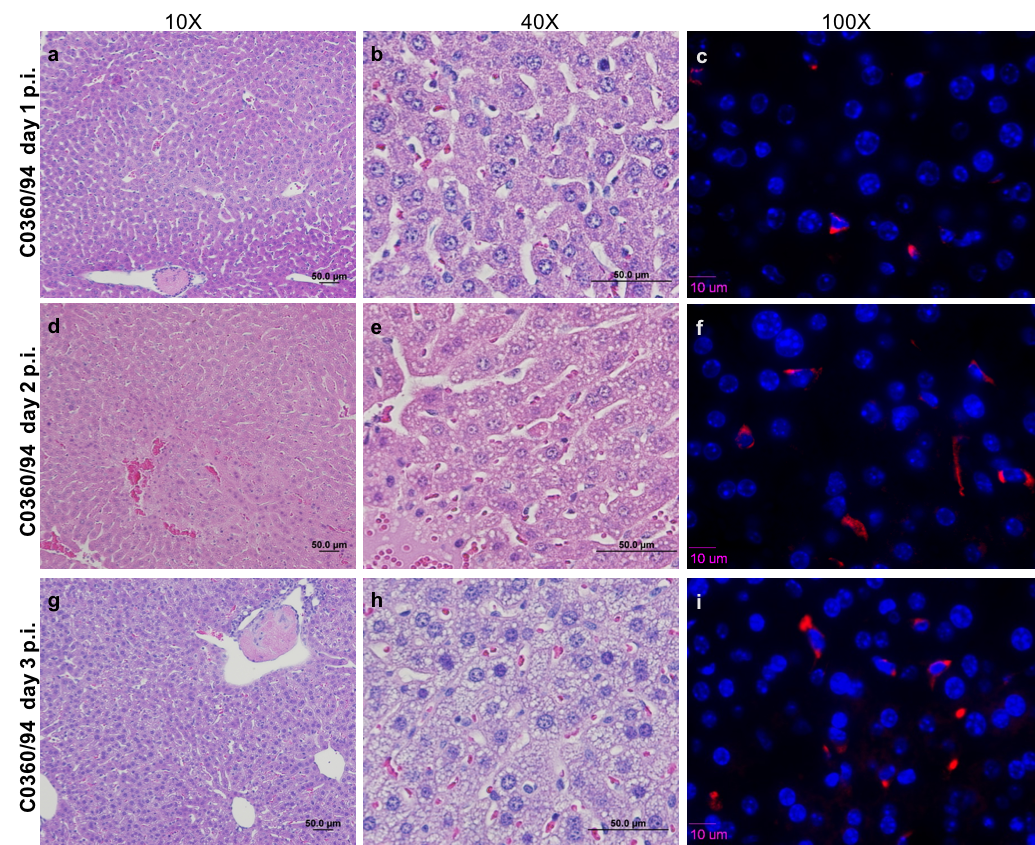


**Supplementary Figure 1:** Histology and immunostaining of liver sections from C0360/94-infected mice. H&E staining of liver sections from animals sacrificed on days 1 (a,b), 2 (d,e), and 3 (g,h) post-infection (p.i.) with a lethal dose of DENV-3 C0360/94 shows damage in the liver sections. 10X (a, d, g) and 40X (b, e, h) magnifications show broad and localized effects on liver tissue. (c, f, i) Immunostaining for DENV NS3 (red) in liver sections indicates that C0360/94 actively replicates in the liver during the course of infection on days 1-3. Blue staining: nuclei, with DAPI.
